# 

**Published:** 1909-11-06

**Authors:** 


### Appointments.

**Dr. James MacKenzie has been appointed an Assistant Physician to the Mount Vernon Hospital for Consumption and Diseases of the Chest, Hampstead and Northwood.**

Swansea Hospital.—**The following appointments have been made:—House Surgeon, Dr. McCormick (at present House Physician); House Physician, Dr. F. W. Waterworth, Sheffield. Additional Anæsthetist, Dr. Alban Evans, Swansea.**

The Council of Sheffield University has made the following appointments:—Mr. George Wilkinson, B.A., M.B., F.R.C.S., to the Lectureship in Diseases of the Ear, Nose, and Throat; and Mr. George H. Pooley, B.A., F.R.C.S. (Eng.), F.R.C.S. (Edin.), to the Lectureship in Ophthalmology.

The first fellowship (established under the will of Dr. Sorby, F.R.S.) has been awarded by the joint committee nominated by the Royal Society and the University of Sheffield to Dr. Jocelyn F. Thorpe, F.R.S. He will engage upon a research on the chemistry of the imino compounds.

Dr. J. C. Mottram, Mr. Bryden Glendinning, and Mr. Somerville Hastings have been reappointed to the "Richard Hollins," "Walter Emden," and "Salters' Company" scholarships respectively for a period of one year in the Middlesex Hospital Cancer Research Laboratories.

Dr. Hughes, First Assistant Medical Superintendent, Lewisham Infirmary, and Assistant Medical Officer of the Workhouse, has resigned, and Dr. W. R. Harris, Second Assistant Medical Officer and Clinical Assistant, has been promoted to the vacant posts.

Mr. D. C. Lloyd-Owen, J.P., has been appointed Consulting Surgeon to the British Ophthalmic Hospital of the Order of St. John of Jerusalem in England, at Jerusalem, vice Mr. R. Brudenell Carter, resigned.

Mr. Herbert Emmerson has been appointed Honorary Ophthalmic Surgeon to the Chesterfield and North Derbyshire Hospital.

Mr. Thomson Henderson has been appointed Surgeon to the Nottingham and Midland Eye Infirmary at Nottingham.

Mr. C. A. S. Dalgleish has been appointed Honorary Surgeon to the Sunderland and County Durham Eye Infirmary at Sunderland, vice Mr. T. F. Hopgood, deceased.

Mr. H. Neville Crowe has been appointed Assistant Surgeon to the Ophthalmic Hospital, Worcester.

### Appointments Vacant.

Greenwich Union Infirmary.—Junior Assistant Medical Officer.

Manchester Children's Hospital.—Out-Patients' Department.—Medical Officer (non-resident).

North Staffordshire Infirmary.—Resident Surgical Officer, House Physician, Junior House Surgeon.

Whitechapel Union Infirmary.—Medical Officer (First Assistant Resident).

### GIFTS AND BEQUESTS.

### EDINBURGH ROYAL INFIRMARY.

The following recent legacies are announced: By the late Mr. John Brown M'Intyre, formerly coffee planter in Ceylon, latterly residing at Villa Aida, 10 Rue Verdi, Nice, France, £500 to account of bequest of £1,000; and by the late Rev. J. B. Atkinson, some time rector of Larling, Norfolk, further payment to account of the residue of his estate—namely, £800. By the late Mr. James Ritchie, formerly of Dilston House, Upper Norwood, Surrey, £2,000 or thereby, to endow a bed to be called the "Catharine Minto Bed," in memory of his wife; and by the late Mrs. Helen Mailer or Stark, Kinross, a one-tenth share of the residue of her estate, probable value £7 or £8.

### SOME OTHER BEQUESTS.

Captain James Chappell, of Glamorgan, has left £500 to the Bridgwater Hospital.

Miss Elizabeth M. Gell, of Douglas, Isle of Man, has left by her will £500 to the Isle of Man Hospital.

Mr. John Newton Whaley, of Lincoln, left by will £200 to the Lincoln County Hospital.

Mr. Thomas Williams, of Scarborough, bequeathed £50 to the Driffield Cottage Hospital.

Mr. Edmund Kell Blyth, of Gresham House, E.C., has bequeathed £100 to the Hampstead General Hospital .

Mr.. James Taddy Friend, of Northdown, Margate, left £100 each to the Margate Cottage Hospital and the Kent and Canterbury Hospital.

As a result of the *Nimrod* exhibition the British Antarctic Expedition, 1907, has sent £25 to the British Lying-in Hospital, Endell Street, W.C.

The Duke of Westminster, out of the money gained for admission to Eaton Hall and gardens, has given £500 to Chester General Infirmary.

Mr. Joseph Powell Swanwick, of Clive House, Winsford, retired physician and surgeon, left estate valued at £16,630 gross, and £12,729 net.

Mrs. Sarah Frances Luard, of Holmhurst, Southborough, left £100 to the National Hospital for Paralysed and Epileptics.

Mr. George Burn, of York House, Hall Green, Worcester, brass-founder, left £200 each to the General, the Queen's, the Women's, the Eye, and the Ear and Throat Hospitals in Birmingham.

Lady (Campbell) Clarke, of Grosvenor Street, W., left £1,000 each to the Westminster Hospital and the Charing Cross Hospital, to endow another bed in the Levy Ward; and £500 to the Hertford Hospital for English people in Paris.

Mr. Alfred Abrahams, of St. John's Wood, diamond merchant, left £500 to the London Hospital, £100 each to the Cancer Hospital, Fulham-road, the Hospital for Diseases of the Heart and Paralysis, the British Lying-In Hospital, the London Fever Hospital (Islington), and St. Mary's Hospital (Paddington).

Mr. Charles Hartley, of Hadham Road, Bishop's Stortford, hon. medical officer to the Bishop's Stortford Hospital, formerly resident medical officer of the Hospital for Consumptives, Ventnor, who died on July 15, aged 60, left estate of the gross value of £10,870 12s. 4d., of which the net personalty has been sworn at £10,727 13s. 2d.

The Tunstall (Staffordshire) Urban District Council has received an intimation that Mr. Edward Malam, a Hanley brewer, has bequeathed to his native town of Tunstall between £30,000 and £40,000 for the purpose of founding a convalescent home for the benefit of the inhabitants.

### THE QUESTION BOX.

**SPECIAL NOTICE.**—Questions of interest affecting the honorary medical staff and hospital administration in all departments are invited. When any point arises we hope our readers will formulate it in a question and so co-operate to make this column of real value and interest. In this way the experience of the best-managed hospitals should be made helpful to the whole hospital world. We shall welcome the contribution of both questions and answers.

**N.B.**—The publication of an answer in this column does not necessarily preclude the publication of subsequent answers to the same question, for a question may involve points which require to be threshed out and fully discussed. Answers, if published, will be paid for at scale rates.

### AN EIGHT HOURS DAY FOR NURSES.

Question: Is the eight hours day for nurses practicable?

ANSWER: Not always.

American Views.

By Dr. Coon, of the Milwaukee County Hospital, and Dr. Hornby, of the Michael Reese Hospital, Chicago.

The question was fully discussed at the ninth annual Congress of the American Hospitals Association held at Chicago in 1907. Dr. Coon, of the Milwaukee County Hospital, thought an eight hours limitation of the period of daily work was desirable, but doubted if it was practicable. At the Milwaukee Hospital the hours of work are from 7 A.M. to 7 P.M. for day, and from 7 P.M. to 7 A.M. for night nurses. Day nurses get one and a half hours for meals and two hours for rest and recreation in between, so that their day is practically eight and a half hours. Night nurses have a full twelve hours stretch. In cases of extreme urgency longer stretches are imperatively necessary and cannot be obviated. Dr. Hornby, of the Michael Reese Hospital at Chicago, said nurses of his hospital had more work than they could very well manage to get through, but they were well paid, and there were no shirkers or grumblers. A twelve hours shift was given in theory; practically nurses got a few hours less, as there were intervals for rest, meals, and recreation. He could not admit that there was any scarcity of nurses or that there was any reason for a scarcity owing to the long hours of service. As a matter of fact there were very many applicants, and a careful selection was always necessary. In the interests of the institutions, as well as of the nurses themselves, it was desirable to make the hours of actual work shorter than they were at present, and he thought that could be managed in most hospitals which possessed a good nursing staff without materially interfering with the practical efficiency of the work.

### HOSPITAL FLOORS AND LINOLEUM.

Question 1. What is the cheapest and most effective detergent for removing ordinary stains and varnish from wooden floors?

ANSWER:

W. J. K. (on the Staff of a Workhouse).

Take 1 part of American pearlash to 3 parts of quick stone lime, slake the lime in water, then add the pearlash, and make the whole about the consistency of paint. Lay the mixture over the whole body of the work which is required to be cleaned with a brush, let it remain 12 or 14 hours, when the stain or varnish can be scraped off. Through large experience I can recommend it.

Question 2. What are the objections to the use of linoleum in hospital wards?

Some Continental Hospitals' Practice.

By W. Milburn, jun.

ANSWER: None when properly laid.

With reference to the query under the above heading, which appeared on p. 113 of your issue of October 23 the following particulars of the flooring to wards in the modern Continental hospitals may be of interest.

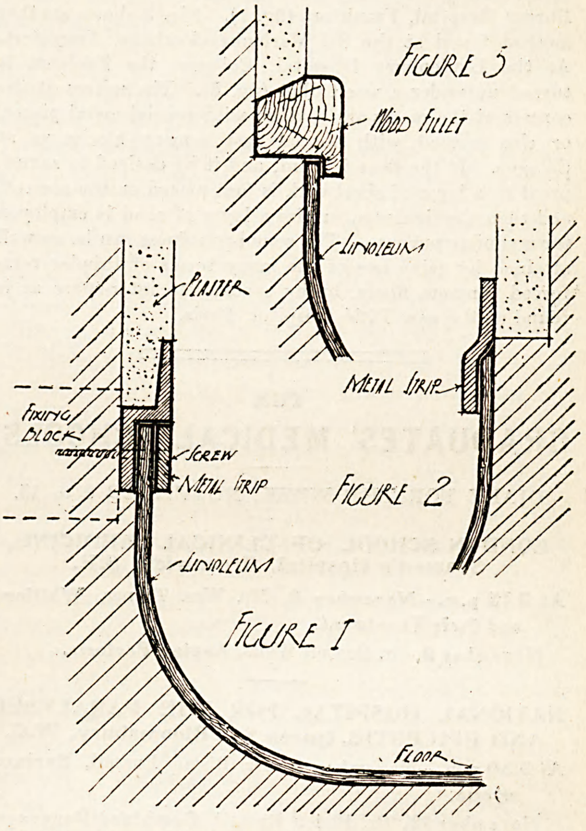

The materials usually employed are tiles, terrazzo, or linoleum. Wood floors are very rare, though wood blocks laid on asphalt are found. The many so-called jointless floors, largely used in Germany a number of years ago on their invention, are now practically discarded owing to their unreliability. Tiled floors are largely employed, as at the Rudolf Virchow and Schöneberg Hospitals, Berlin, the St. Georg Hospital, Hamburg, Nuremberg, Utrecht, and Paris. When properly laid they make an excellent floor, though perhaps somewhat cold for general adoption in England. Terrazzo, again, is largely found, as at Charlottenberg and the Eppendorf Hospital, Hamburg. It should be laid in slabs some 10 feet square, separated by brass slips to guard against the cracks, which inevitably occur when it is laid in large sheets.

Linoleum is now being very largely adopted on the Continent, and is found in the Frankfort Bürger and Municipal Hospitals, the Third Hospital and the Ophthalmic and Psychiatrical Clinics, Munich, Dusseldorf General Hospital, and the Lindenburg Hospital, Cologne. When properly laid it is practically jointless, hard, durable, warm, sanitary, and easy to clean. It should never be laid on wood floors, particularly the ground floor, as, owing to its impervious nature, any moisture rising is deposited on its under side, and, if the ventilation is poor, "dry rot" speedily attacks the timber. Wood-joisted floors should not, however, be found in a modern hospital—fireproof floors only should be employed. The surface of the floor should be screeded smooth in cement, and on this the linoleum should be glued down or attached with shellac, as at the Johannstadt Hospital, Dresden. The joints between the strips should be made with liquid indiarubber. The principal difficulty is the formation of the rounded angle between wall and floor, as the common method of finishing the linoleum on the flat against an angle of wood or other material is unsatisfactory, leaving as it does a joint for dirt to get in. The angle should first be rounded in cement and the linoleum turned up the wall to a height of about 6 inches, the upper edge being then securely fastened with a special metal clip, as at the Bürger Hospital, Frankfort (fig. 1). Fig. 2 shows another method found at the St. Marienkrankenhaus, Frankfort. At the Lindenburg Hospital, Cologne, the linoleum is turned up under a wood fillet (fig. 3). The mitres at the corners of the rooms are covered with special metal pieces, or else formed with special cast cement blocks as at Cologne. If the floor is solid, and it be desired to sound-proof it, a layer of sheet cork is first placed on the cement, and then the linoleum, or else a layer of sand is employed for a similar purpose. The sound-proofness can be as well attained by using one of the many forms of tubular reinforced concrete floors, or by a complete inter-space as is found at the new Pitié Hospital, Paris.

### THE GRADUATES' MEDICAL SCHOOLS.

DIARY FOR THE WEEK, NOVEMBER 8 to 13.

### LONDON SCHOOL OF CLINICAL MEDICINE, Seamen's Hospital, Greenwich, S.E.

**At 3.15 p.m.—November 8**, Mr. Wm. Turner, **Whitlows and their Treatment.**

**November 9**, Dr. Russell Wells, **Angina Pectoris.**

### NATIONAL HOSPITAL FOR THE PARALYSED AND EPILEPTIC, Queen Sq., Bloomsbury, W.C.

**At 3.30 p.m.—November 9**, Dr. Risien Russell, **Syringomyelia.**

**November 12**, Dr. Risien Russell, **Combined Degeneration of the Cord.**

### MEDICAL GRADUATES' COLLEGE AND POLYCLINIC, 22 Chenies Street, W.C.

**At 4 p.m.—November 8**, Dr. H. G. Adamson, **Skin.**

**At 5.15 p.m.—November 8**, Mr. A. H. Tubby, **Surgical Diseases of Children** (continued).

**At 4 p.m.—November 9**, Dr. Frederick Taylor, **Medical.**

**At 5.15 p.m.—November 9**, Dr. T. D. Lister, **Lung Affections after Injuries.**

**At 4 p.m.—November 10**, Mr. P. J. Freyer, **Surgical.**

**At 5.15 p.m.—November 10**, Mr. H. Stansfield Collier, **The Surgical Complications of the Inflammations of the Colon.**

**At 4 p.m.—November 11**, Sir Jonathan Hutchinson, **Surgical.**

**At 5.15 p.m.—November 11**, Dr. Lewis Smith, **The Treatment of a Failing Heart.**

**At 4 p.m.—November 12**, Dr. H. Tilley, **Ear, Nose, and Throat.**

### NORTH-EAST LONDON POST-GRADUATE COLLEGE, Prince of Wales's General Hospital, Tottenham, N.

**At 4.30 p.m.—November 9**, Mr. Henslowe-Wellington, **Forensic Medicine and Coroner's Law.**

### ST. JOHN'S HOSPITAL FOR DISEASES OF THE SKIN, Leicester Square, W.C.

Chesterfield Lectures.—At the Hospital on Thursday evenings at 6 p.m., each lecture followed by clinical demonstrations :—

**November 11, Syphilis** (continued): *Papular* (I., Miliary; II., Lenticular; III., Squamous; IV., Moist), *Pustular* and *Tuberculous*.

### THE POST-GRADUATE COLLEGE, West London Hospital, Hammersmith, W.

**At 10 a.m.—November 8** and **11**, Surgical Registrar **Demonstration.**

**November 12**, Medical Registrar, **Demonstration.**

**At 12 noon.—November 11**, Dr. Bernstein, **Pathological Demonstration.**

**At 12.15 p.m.—November 9**, Dr. Pritchard, **Practical Medicine.**

**November 10** and **13**, Dr. Grainger Stewart, **Practical Medicine.**

**At 5 p.m.—November 8**, Dr. Davis, **Otorrhœa.**

**November 9**, Dr. Moullin, **Gynæcological Cases.**

**November 10**, Dr. Beddard, **Medicine.**

**November 11**, Mr. Dunn, **So-called "Second Sight": its Features and Causes.**

**November 12**, Mr. Bidwell, **Clinical Lecture.**

